# Adaptive ecological knowledge among the Ndjuka Maroons of French Guiana; a case study of two ‘invasive species’: *Melaleuca quinquenervia* and *Acacia mangium*

**DOI:** 10.1186/s13002-023-00602-7

**Published:** 2023-07-11

**Authors:** Johanna Theys, Marc-Alexandre Tareau, Clarisse Ansoe-Tareau, Alexander Greene, Marianne Palisse, Alizée Ricardou, Guillaume Odonne

**Affiliations:** 1 Laboratoire Ecologie, Evolution, Interactions des Systèmes amazoniens (LEEISA), CNRS, Université de Guyane-IFREMER, 97300 Cayenne, French Guiana; 2 Groupe d’Etude et de Protection des Oiseaux en Guyane (GEPOG), Remire-Montjoly, French Guiana; 3 CIC INSERM 1424, Clinical Investigation Center, Cayenne General Hospital, Cayenne, French Guiana; 4 Interpreter-Translator in Surinamese Maroon Languages, Okanisi Traduction et Médiation, Remire-Montjoly, French Guiana

**Keywords:** Invasive species, Maroons, Savannas, Local ecological knowledge, Biocultural interactions, Environmental perceptions

## Abstract

**Background:**

To understand how local ecological knowledge changes and adapts, here in the case of the recent introduction of plant species, we report the knowledge and perceptions of the Ndjuka (Maroon) of French Guiana concerning two tree species, *Acacia mangium* and *niaouli* (*Melaleuca quinquenervia*), which are categorized as “invasive alien plants” in the savannas of their territory.

**Methods:**

To this end, semi-structured interviews were conducted between April and July 2022, using a pre-designed questionnaire, plant samples and photographs. The uses, local ecological knowledge, and representations of these species were surveyed among populations of Maroon origin in western French Guiana. All responses to closed questions collected during the field survey were compiled into an Excel spreadsheet in order to perform quantitative analyses, including the calculation of use reports (URs).

**Results:**

It appears that the local populations have integrated these two plant species, which are named, used and even traded, into their knowledge systems. On the other hand, neither foreignness nor invasiveness seem to be relevant concepts in the perspective of the informants. The usefulness of these plants is the determining factor of their integration into the Ndjuka medicinal flora, thus resulting in the adaptation of their local ecological knowledge.

**Conclusion:**

In addition to highlighting the need for the integration of the discourse of local stakeholders into the management of "invasive alien species,” this study also allows us to observe the forms of adaptation that are set in motion by the arrival of a new species, particularly within populations that are themselves the result of recent migrations. Our results furthermore indicate that such adaptations of local ecological knowledge can occur very quickly.

**Supplementary Information:**

The online version contains supplementary material available at 10.1186/s13002-023-00602-7.

## Background

French Guiana is largely covered by tropical rainforest [1]. However, coastal savannas, which occupy only 0.3% of the French Guianese territory, hold approximatively 16% of the French Guianese flora and contain many emblematic, rare, or threatened fauna [2, 3]. The savannas of the French Guianese coast are considered a dynamic cultural landscape due to ancient human influences [4–7]. Today, the vast majority of the French Guianese population lives in the coastal plain, many in close proximity to this ecosystem, which leads to multiple pressures such as infrastructure development, urbanization, agricultural use, sand mining, uncontrolled fires and introduced species [6]. These observations have led to an increase in scientific interest and studies on Guianese savannas, which raise important conservation issues [8, 9]. Among the invasive alien species (IAS) present in French Guiana, *Acacia mangium* Wild. (Fabaceae) and *Melaleuca quinquenervia* (Cav.) S.T.Blake (Myrtaceae) have been presented as the most problematic ones considering their widespread distribution and capabilities for environmental transformation of the savanna ecosystem [8, 10].

Regarding the first species, *A. mangium* is a fast growing, pyrophytic, evergreen tree up to 30 m [8], which was introduced for agronomic trials by the French International Research Center for Agronomy and Development (CIRAD) as part of the “Plan Vert,” a 1970s plan to develop French Guianese economy, which included industrial and agricultural components [11]. CIRAD was searching for plants with potential for wood or fodder production. It was introduced again in the 1990s–2000s by the French Forest Bureau (ONF) and CIRAD with the aim of reforesting mining sites [11]. This species, originating from Australia and Indonesia, is known to pose conservation issues in most places where it has been introduced such as the Dominican Republic or various Pacific islands [12]. *Melaleuca quinquenervia* (a.k.a. *niaouli*) is an evergreen tree growing up to 10 m in swampy areas, which originated from eastern Australia, Indonesia and New Caledonia [13]. When and for what purpose it was first introduced to French Guiana are unknown, although the plant’s presence was first recorded in 1948 (herbarium specimen n°4349 of the Cayenne herbarium-CAY). In the 1980s, *niaouli* was introduced once again as part of the Plan Vert in order to develop the paper industry [8].

Plant introduction is not a recent phenomenon, since plants have been carried by humans on their journeys for thousands of years [14]. In fact, the majority of plant introductions over the last 500 years are of anthropogenic origin, largely because the introduction of plants has been a major phenomenon of colonization that has increased even more rapidly since the end of World War II [15–17]. Conversely, the concept of IAS (https://www.cbd.int/invasive/WhatareIAS.shtml) is much more recent, as it was first proposed by the zoologist C. Elton in 1958 [18]. During the 1990s, the concept developed into a subfield of biology and was gradually incorporated into environmental conventions as a key issue on which action is needed. Since then, invasive species management and control programs supported by public policies, scientists and environmentalists have emerged [19] in parallel with the increasing interest in biodiversity [20].

However, recent studies have made the subject more controversial [19, 21–23], questioning, for example, the claim that invasive species are the second most significant cause of biodiversity loss. Others have interrogated the concepts of ‘alien’ or ‘invasive,’ categories put forward as universal but which are rather contextual [24–26]. The scientific debate brings up the multiplicity of perceptions surrounding invasive species [22, 27], which we explore here in order to ground the concerns of conservation ecologists with other opinions [23]. Such alternative perceptions have become a focus of ethnobotanical research in recent years, using the cultural relativity of relationships to the environment or the existence of various ontologies [28] as drivers that could explain other ways of perceiving plant introduction. Building upon recent anthropological perspectives such as multispecies interactions [29], we employ these two species to shed light on neglected aspects of human–plant relationships.

In a context where plant knowledge changes/adapts through space and time for a multiplicity of reasons, including human migration [30–33], geopolitics [34–36], economic factors [37], urbanization [38, 39] and cultural encounters [7] (some of these parameters obviously overlap), the adaptation of local ecological knowledge to alien species remains controversial. Studies have described the arrival of new species as culturally impoverishing, especially to populations already exposed to the erosion of their knowledge [30, 40]. In extreme situations, these introductions can even threaten the way of life and subsistence capacities of certain populations, as is the case for Wapishana and Makushi communities in the Brazilian northern Amazon regarding the introduction of *A. mangium* to their Indigenous territories by forestry companies [41].

In other instances, IAS have been included into local practices, becoming culturally enriching [42, 43]. While they appear invasive or harmful to some cultural groups, they are included in traditional plant-based medicinal practices by others [44]. Some of these species are so deeply incorporated into local practices that they are even considered native by their users [45], such as the domestic buffalo (*Bubalus bubalis*) for the Jawoyn aboriginal community in the Australian national park of Kakadu [46]. Widespread species found in both the countries of departure and arrival of migrating populations can also be described as culturally facilitating [45], for instance when a so-called “traditional” pharmacopoeia is reconstructed in a host territory thanks to globalized plants [28]. The diversification hypothesis even suggests that IAS could complement lacking uses, thus supporting cultural evolution [47, 48]. Other studies also suggest that the use of IAS by local populations could also reduce the use pressure on native species [49].

### Aim of the study

The aim of this study is to document adaptive mechanisms of local ecological knowledge and social responses to plant introduction within the framework of a socio-environmental perspective. More specifically, we aim to question the relationships that mobile societies establish with plants which are themselves mobile. We further aim to problematize a controversial subject in the field of ecology in order to bring new elements of reflection to environmental management practices. The concept of IAS is the subject of scientific debate, and this article extends that debate and proposes avenues of thinking about the relations between humans and non-humans in dynamic contexts. The purpose of this article is therefore to contribute to the discussion on this controversial subject by providing a new perspective through a field approach.

**Fig. 1:**
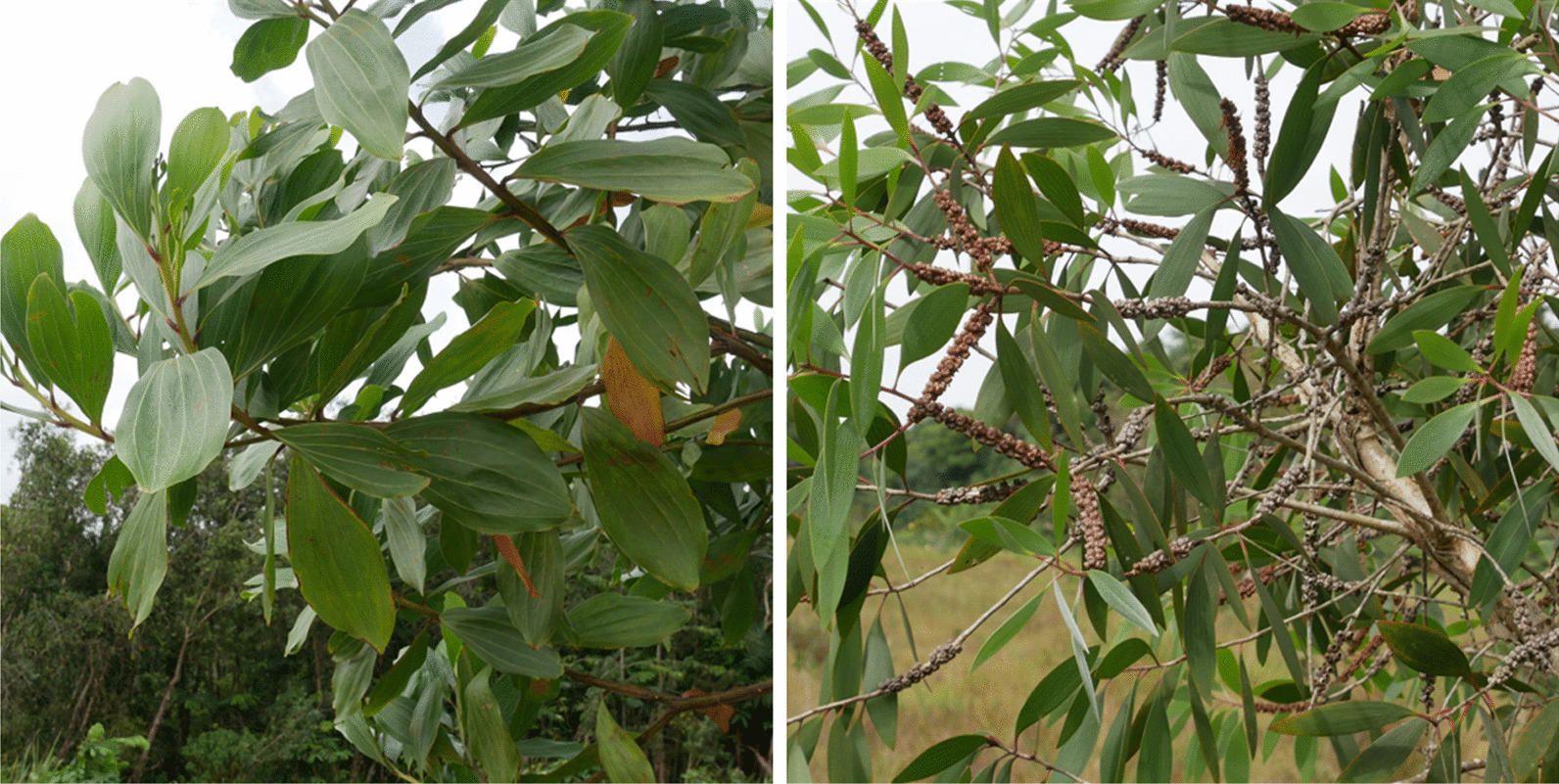
Respectively, pictures of an Acacia mangium branches and a *Melaleuca quinquenervia* one

We have chosen to emphasize the cultural mosaic and the significant human mobility that characterize French Guiana in order to study the relationship between the territory's inhabitants and invasive species. To do so, we focus on the Maroon population, a series of cultures formed by the descendants of escaped slaves during the colonial period, which has recently immigrated to and is in the process of settling and appropriating territory in western French Guiana, mainly due to the Surinamese civil war that took place between 1986 and 1992. Among the Maroon peoples, four are present in French Guiana: The Saamaka, the Paamaka, the Aluku and the Ndjuka. The Ndjuka (a.k.a. Aukan, Okanisi) escaped from plantations during the seventeenth and eighteenth centuries and resettled around the Tapanahoni River basin in Suriname, which they today consider as their cultural origin [50]. Ndjuka people are thought to number around 47,000 in French Guiana, of which 33,500 are located in the coastal region [51].Our primary goal is to determine what links Ndjuka Maroons may have established with the two species considered to be the most problematic for the conservation of Guianese savannas, namely *A. mangium* and *niaouli* (Fig. 1). Ndjuka Maroons represent a segment of the French Guianese population that is often economically and administratively precarious; many are in an irregular situation and do not receive any social aid. Combining various livelihood activities such as hunting, fishing and gathering is therefore an important part of their way of life. As this population is not well represented in the media, institutions, or political bodies, their views on environmental changes are thus poorly known.

## Methods

### Field sites

**Fig. 2:**
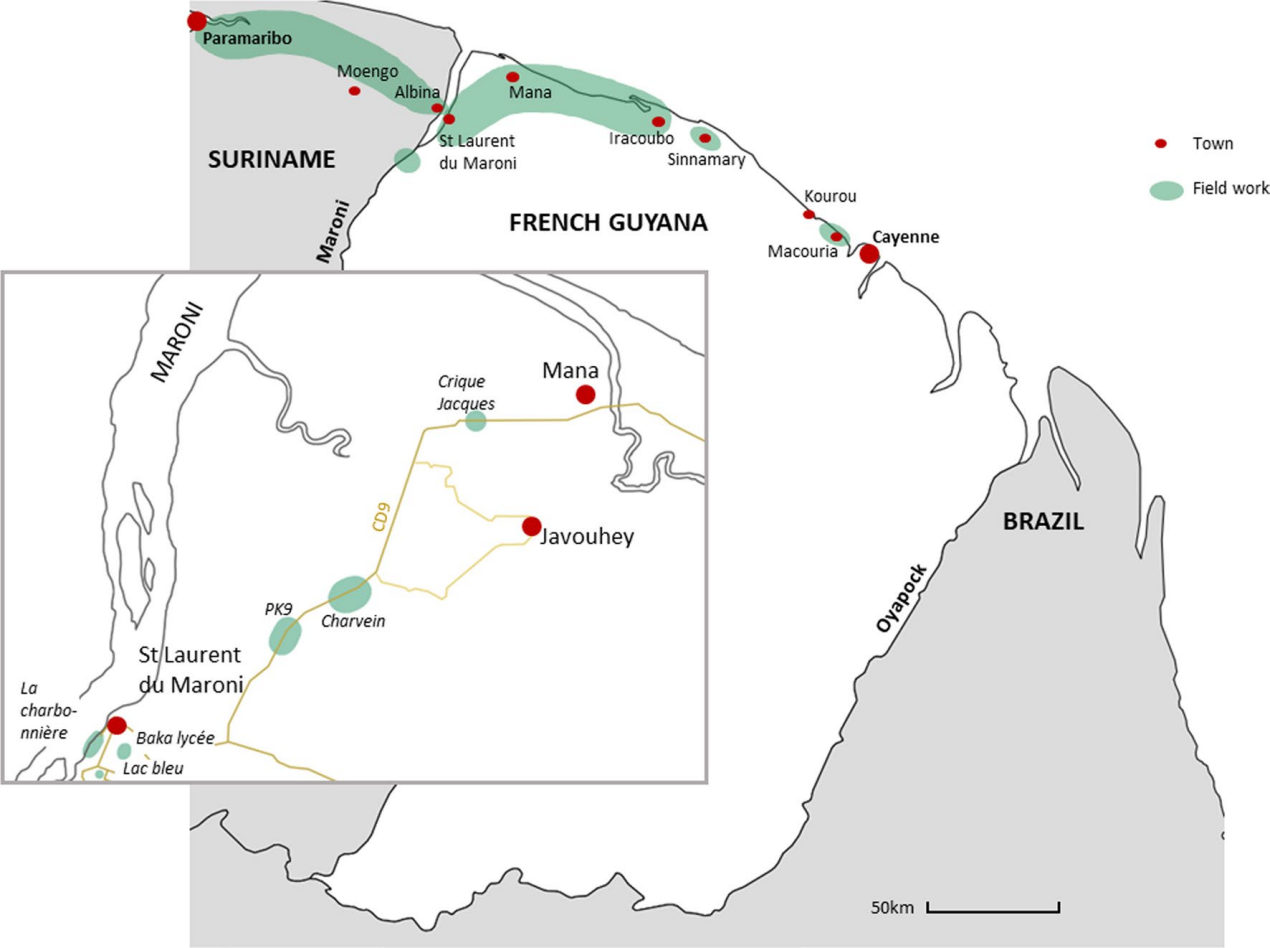
Map of the field sites

Fieldwork mainly took place in western French Guiana between the towns of Mana and Saint-Laurent-du-Maroni during 4 months between April and July 2022. Occasional field sessions were carried out outside this area around the towns of Sinnamary and Iracoubo in order to complete the data set. A further field mission was also carried out in Suriname between Albina and Paramaribo to clarify some of the questions that arose during the survey (Fig. 2).

### Data collection

The first people interviewed were approached informally, on roadsides, in farmer’s markets (Saint-Laurent-du-Maroni, Mana, Albina), at harvesting areas or at their workplaces. The presence of *pikin osu*(the Nengee tongo name of roadside stalls to sell cassava juice –*kasaba wataa*-, fruits and vegetables, wooden sculptures, etc.) along the CD8 and CD9 roads linking Mana to Saint-Laurent-du-Maroni was an important indicator of key locations to conduct surveys. As the size of the population is unknown, we then used non-random sampling techniques (accidental, convenience or snowball sampling [52]) to gradually contact respondents. After obtaining individual prior informed consent (Additional file 1), semi-structured interviews were conducted using a pre-designed questionnaire (Additional file 2) structured around four distinct topics:

- Local ecological knowledge of both plants
- Uses and means of supply
- Perception and representation regarding both plants’ behaviors
- Perception of invasive alien species management actions by environmentalists

Interviews were conducted in French or with the help of a translator in Maroon languages (Nengee tongo, Saramaka tongo) and Surinamese Creole (Sranan tongo). All quotations in the text have been translated by the authors.

Data were also collected in focus group discussions, using participant observation and visual stimuli [52, 53], which consisted of photographs of the species studied at different stages of maturity and of different plant parts (seed, flower, trunk, etc.) as well as fresh plant samples (branches without seeds).

### Data analysis

All responses to closed questions collected during the survey were sorted, categorized and compiled in an Excel spreadsheet in order to carry out thematic and quantitative analyses, which included the calculation of use reports (URs), defined as the frequency of citation of a species or its specific uses [53].

## Results

### People interviewed

A total of 56 interviews were conducted using semi-structured questionnaires. Of these, 11 interviewees (20%) did not identify themselves as of Maroon origin and were as a result not included in the data analysis. Among the 45 remaining interviews (80%), people identified themselves as of Maroon origin, mostly Ndjuka (78%, *N* = 35) or Saamaka (20%, *N* = 9) and only 2% Aluku (*N* = 1). Some interviewees mentioned belonging to several cultural groups but identified themselves primarily with their mother's group, since these cultures are based on matrilineal kinship systems.

Within the 45 interviews, 43 represent individual interviews whereas the remaining two are group interviews, whether of family, neighbors, or common interest. Each group interview has been considered as a single interview in which information provided by different members of the group have been combined (two to six people).

The overall group contains 49% women (*N* = 21) and 51% men (*N* = 22), and 41% of respondents stated that they were born in French Guiana compared to 59% in Suriname. At the time of the study, informants were between 13 and 65 years old, with an average of 38.7 years. Twelve were between the age of 13 and 30, 20 were 30 to 50, and 11 were 50 to 65 years old. Gender, birthplace, and age in group interviews were not recorded.

Informants were involved in various types of activities and daily practices. Most of them carried out subsistence activities such as hunting, fishing and growing food and medicinal plants, and are part of an informal economy through roadside and market sales, handicrafts, or odd jobs.

### Local ecological knowledge regarding *Acacia mangium*

*Acacia mangium* was recognized by 71% (*N* = 32) of the informants; however, very few were able to provide names for it in Nengee tongo and Saamaka tongo languages (Table 1). Twenty-five (56%) respondents were able to indicate geographical locations where the plant grows. Only three (7%) mentioned places outside French Guiana, namely Suriname, Haiti and Brazil.

**Table 1:** Local names for *Acacia mangium*

Name mentioned	Citations	Language spoken^a^
*Mila bon*	3	Nengee tongo
*Sumake*	1	Saama katongo
*Sukru bon*	1	Nengee tongo
*Yarakopi*^b^	1	Saamaka tongo
*Acacia mangium*	1	Nengee tongo

^a^Languages spoken on the field are multiple. People practice code-switching, meaning that they change languages in conversation. As a result, some terms may be present in several languages, making the affiliation of the different nominations to a particular language difficult

^b^This name normally refers to an unrelated plant, *Siparuna guianensis*, and is thus likely a misidentification on the part of the interviewee

Various ecological features were reported by the interviewees, with the plant’s rapid growth and seed dispersal as the most mentioned (24 responses; 53%). The fact that it attracts red ants (4 responses; 9%) and likes to grow near water were also characteristics, among others, mentioned several times.

Overall, 13 people (29%) reported using the plant for personal use. Interviews indicated 18 different uses of the plant (Additional file 3). Charcoal, firewood (especially for cooking), soil amendment and building timber were among the most common uses. *A. mangium* is also used for medicinal baths, mainly to cure fever and treat muscle soreness.

These results indicate that *A. mangium* is not often used, and when it is used, it is for opportunistic or proximal purposes. Supplies are obtained by collecting branches from the roadside and by cutting down trees when they are close to the house. Moreover, no mention of selling or buying the plant was made or observed during the study.

### Local ecological knowledge regarding *Melaleuca quinquenervia*

Almost all of the people interviewed (93%; *N* = 42) recognized the *niaouli* plant during the survey. Respondents indicated six names of the plant in Nengee tongo, Saamaka, French and Guianese Creole languages (Table 2).

**Table 2:** Local names for *Melaleuca quinquenervia*

Name mentioned	Citations	Language spoken
*Fekisi uwii*	30	Nengee tongo
*Albina uman*	22	Nengee tongo
*Fey mant*	1	French Guianese creole
*Andoja*	1	Saamaka tongo
*Pikin tiki*	1	Nengee tongo
*Niaouli*	1	French

Many of the people interviewed (58%; *N* = 26) were able to indicate where the plant grows geographically. Unlike *A. mangium*, which was only observed by respondents occasionally outside of French Guiana, *niaouli* is well known to thrive beyond its borders, especially across the Maroni River in Suriname (29 responses; 64%), around the towns of Albina (16 responses; 36%) and Moengo (4 responses; 9%).

When asked about their ecological knowledge of the plant, many noted its fast growth (6 responses; 13%) and preference for swampy areas (6 responses; 13%). Others noted that the tree is especially common in anthropogenic, disturbed areas.

*Niaouli* is used, more or less regularly, by 17 of the 45 people interviewed (38%). Twenty-two uses were recorded (Additional file 4), however by far the most common is as an ingredient of genital steam baths.

### Supply methods and distribution network of *Melaleuca quinquenervia*

**Fig. 3:**
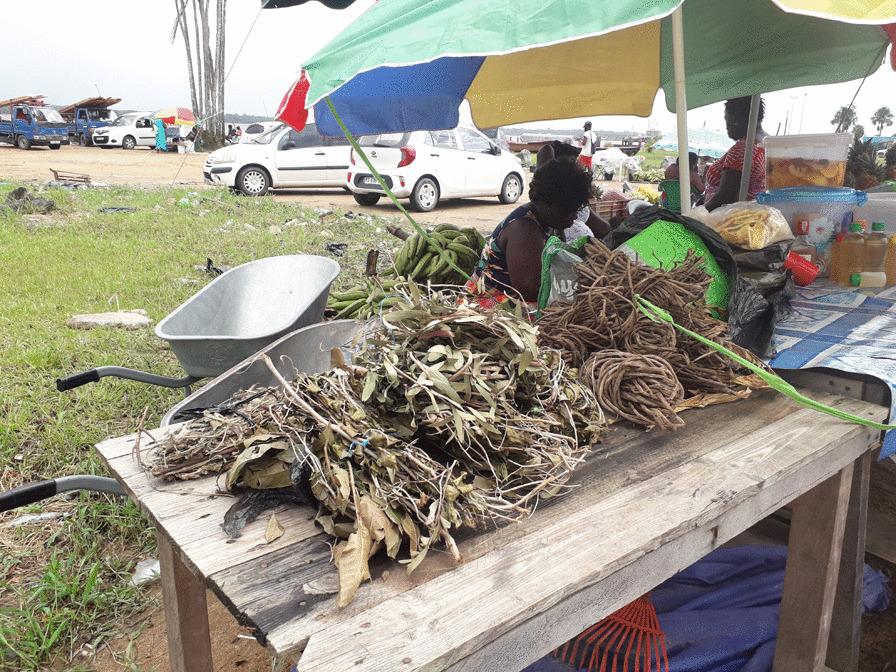
Bundles of *Melaleuca quinquenervia* leaves for sale in “la charbonnière”, a Maroon neighborhood in Saint-Laurent du Maroni

Most informants obtain the plant by harvesting it (24 URs; 53%). After a birth, men of the family are sent to collect the plant, as need for the plant is highest during the post-partum period. People often cross the Maroni River to get it from the town of Albina (Suriname), where the *niaouli* is known to be widely available. It is also possible to find the plant sold as bundles of branches and leaves in markets in western French Guiana (Fig. 3), and even more commonly in Suriname.

**Fig. 4:**
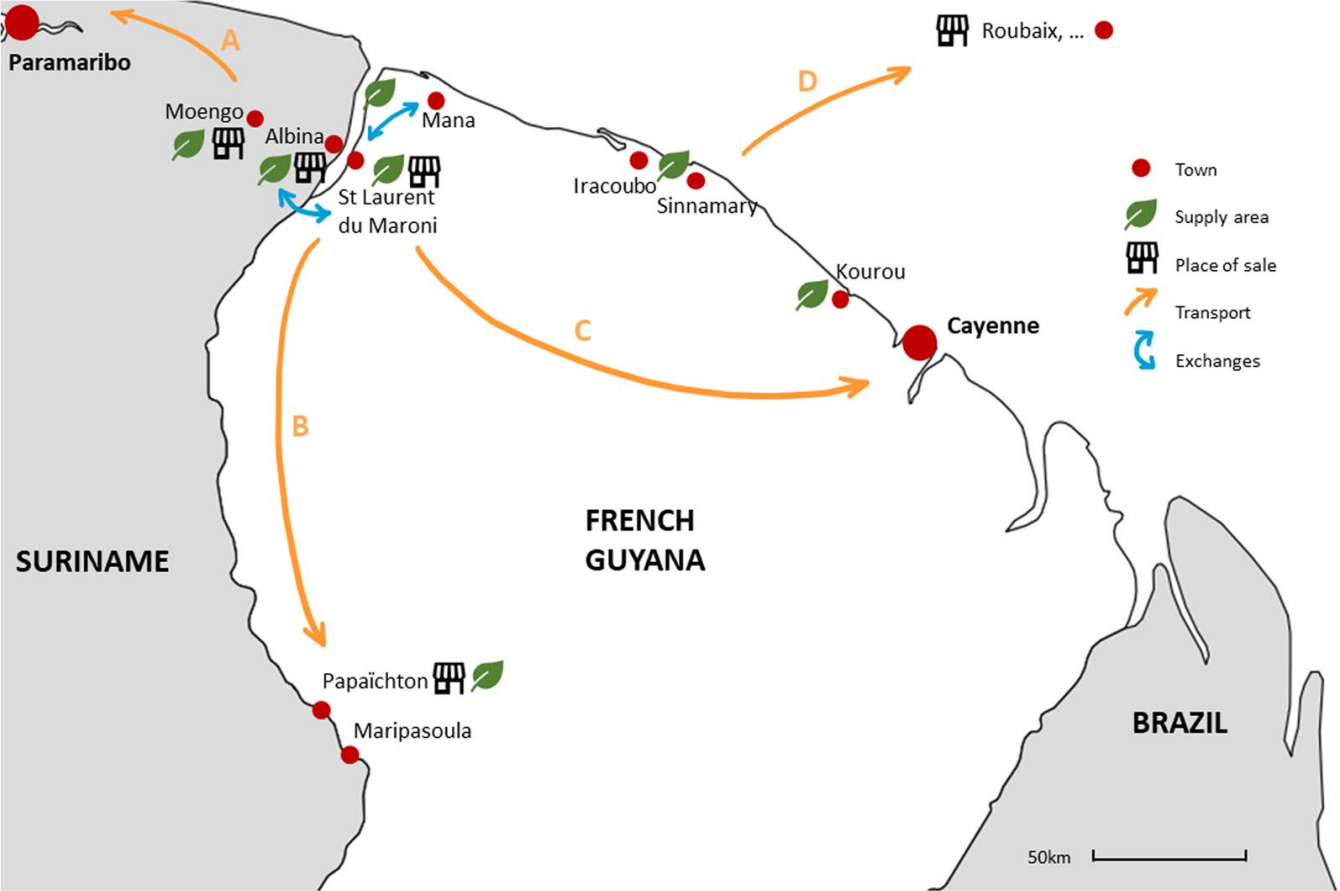
Circulation map of *Melaleuca quinquenervia* in the study area (**A** Delivery of bundles to sellers at Paramaribo's plant market; **B** transport to inland towns by canoe; **C** transport by West Guyanese traveling periodically to Cayenne; **D** dried plants sent by mail to families in the French mainland)

Several interviewees explained that *niaouli* is planted by some people in their gardens, especially when they are located far from the plant’s area of distribution. The supply chain of *niaouli* reported by respondents is mapped in Fig. 4, which shows the primary axes of circulation to be east–west along the Surinamese-French Guianese coast and north–south along the Maroni River (Fig. 4).

### Perceptions of foreignness or invasiveness

*A. mangium*was considered native by 12 respondents (27%). The few who mentioned it being introduced to the country (5 responses; 11%) were aware of the involvement of agronomic institutions, although the country of origin was unknown. Similarly, *M. quinquenervia* is considered native to the French Guianese territory by 16 informants (36%). Only three interviewees (7%) brought up the idea of a non-native origin of *M. quinquenervia*. For others, the idea of a human-mediated introduction of the plant to the territory is in fundamental opposition to their beliefs:

“God created Earth, there are plants we use, other we do not, but each thing has its place […] If God has put all this greenness here, there must be a reason.”^1^Ndjuka man, 65 years old, Charvein.

“Because for us, in our animistic culture, everything that is in a place, was predicted to be there. So, stating that someone brought it here, is like going against our beliefs.”^2^Ndjuka woman, 27 years old, Mana

The concept of 'invasive' made little sense to the interviewees, as for many, it had no meaning in their cosmologies. Few were able to define it as the idea of a plant taking over too much space, or even that of other species. Nevertheless, the concept was not always perceived as negative, as the plants have uses or grow in areas that are not easily habitable or exploitable. Some mentioned the idea of a human invasion into the plant’s territory rather than the other way around.

Overall, interviewees view favorably the presence of both plants (14 responses; 31%), mainly because of their uses or because of the belief that every plant has a purpose (“*ala uwii bun”—*every plant is good). However, some people still have reservations and concerns about *A. mangium,* as it takes over some agricultural fields and attracts red ants, which represent a danger for children. It was reported that in the worst cases, farmers have had to leave their fields. Several techniques to get rid of the plant when it becomes inconvenient were mentioned, such as girdling, logging, burning, grinding it up or using chemicals.

### Consideration of environmental management methods toward invasive species

When environmental management methods (control and eradication) of IAS were presented to informants, 57% were opposed to them when it came to *Niaouli* and 50% for *A. mangium*, arguing that the plants have several uses and are already integrated into local knowledge and practices. In addition, to many, these plants along with others provide an alternative to biomedicine, and are freely available resources easy to harvest along the roadsides. Among the various reactions, some believed that control of IAS is impossible: “If they cut it, it will regrow like hair,” (Ndjuka man, 56 years old, Charvein), while others questioned the urgency and necessity of these measures.

To other informants, environmental managers are just not aware of the uses of the plants. Some also challenged the legitimacy of external environmental managers to intervene on land occupied by locals, who are not part of the decision-making process and feel deprived of their rights to participate. One of the most frequent reactions was to ask for more consultation and dialog with local populations that wish to be integrated into management discussions: “People that live here must be integrated [into the management process]” (Ndjuka man, 40 years old, Mana). Some people are even considering "disobedience" actions, for instance buying the plants from Suriname or planting them on their own land.

## Discussion

Given the nearly twenty uses reported for the two plants studied, multiple folk names and even the development of a distribution network, the rural Ndjuka populations of western French Guiana, themselves recent migrants, seem to have largely taken ownership of these species.

### Plant names as testimonies of an adaptive process

The process of naming a new species is always significant, and gives insight into the relations a cultural group has with non-humans [54], or with humans from other cultural groups [55]. Linguistic legacies from Africa were for example used when enslaved Africans adapted their knowledge to medicinal floras in the Americas [56, 57]. The small number of names, along with their low citation counts, for *A. mangium* can be interpreted as a modest interest for this species. Nevertheless, the few names given to this tree indicate a close observation of the ecological features of the plant*. Mila bon*, for instance, means “ant tree,” as inhabitants noticed that red ants were particularly attracted to the plant. Likewise, *sukru bon*, meaning “sugar tree” was employed because of its fertilizing properties to the soil that would “feed the soil, give it sugar.” *A. mangium* being a Fabaceae, it fixes nitrogen into the soil, explaining the fertilizing effects noticed by the inhabitants.

On the other hand, the numerous citations for *M. quinquenervia* reflects a true interest. The highest cited name, *fekisi uwii*, relates to the pharmaceutical Vicks® unguent (called *fekisi*) due to the similar smell, which has led to the same use. *Albina uman*, which means “the woman from Albina,” testifies to one of the main collection places of this species (from the Paramaribo perspective) and to the fact that it is primarily a women’s plant [44]. Lastly, *andoja* is an interesting loan name that usually stands for another aromatic Myrtaceae, *Campomanesia aromatica* (Aubl.) Griseb. [58], which was among the most sold plants for vaginal steam baths in the Paramaribo market a decade ago [59].

### *Niaouli* as a recent “must have” for women’s vaginal baths

*Niaouli* has been reported by recent ethnobotanical studies [7] for use in vaginal steam baths, but it is surprisingly absent from similar older studies [59, 60], which suggests that its inclusion in women's baths is in fact relatively recent, possibly as a substitute for *Campomanesia aromatica*.

**Fig. 5:**
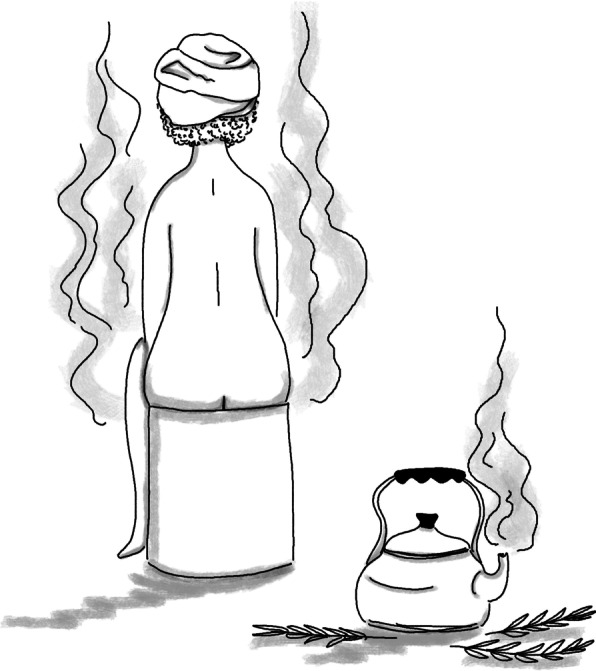
Woman steam bathing

These plant-based steam baths are generally practiced twice a day by Maroon women, once they have “received the *pangi”* (adulthood ritual which consists in offering a piece of cloth, a *pangi*, to a young woman along with advice for a woman’s daily life) or after their first childbirth. Water is boiled in a *ketee* (large metal teapot), to which *niaouli* leaves and/or other plants are added. The decoction is then poured into a *boketi* or a *dodo* (containers of large and medium sizes), on which women sit to wash and steam [44] for intimate hygiene (Fig. 5). These baths are practiced as a daily routine or after childbirth, as they help to remove remaining blood and to “smell good.” They aim to 'close' the vagina to prevent the 'cold' from entering. 'Closing' or 'tightening' the vagina is also practiced to increase the sexual pleasure of the partner (dry sex method). Ultimately, steam bathing cleanses the impurities represented by menstruation in Maroon cultures, and thus avoids affecting the husband’s *obia* (allied non-human spirits in Nengee tongo) [59–61].

This species is known for the high content of essential oil in its leaves, including (depending on the chemotype) *E*-nerolidol, linalool, 1, 8-cineole, viridiflorol, α-terpineol and β-caryophyllene[62]. Its essential oil has been shown to have antimicrobial activity against a large range of microorganisms, such as *Staphylococcus aureus* (MIC = 100 μg/ml) [63], drug resistant *Candida auris* (13.3 mm inhibition/agar disk diffusion method) [60], *Candida glabrata*, *Candida albicans*, *Candida krusei*, and *Candida guilliermondii* (respectively 16, 12.8, 12, 17.3 mm inhibition/agar disk diffusion method) [64, 65]. The four last species are notably involved in vaginal candidiasis.

### An integration related to usefulness?

The reported knowledge and uses are not homogeneous across the informants. Although this could be a consequence of our sampling methodology, we suggest that it might also vary according to several conditions, including geographical distance from the plant, cultural group, origin, history of migration, age and livelihood activity. Similar examples of the integration of species considered invasive into local practices have been observed in recent ethnobotanical studies, showing that this is not an unusual phenomenon [40, 42, 48, 66]. For instance, the study conducted by Dos Santos (2014) shows that the Minguiriba and Riachao populations of Northern Brazil consider most plants categorized as invasive, useful, mainly for fodder or medicinal purposes. But under which conditions and through what processes does a plant become useful? Are populations in a migratory process more likely to adapt or even appropriate species that have recently been introduced to the territory? Leonti’s hypothesis of a “prototypical medicinal plant” [67], would predict that *niaouli*, with its notable organoleptic properties, is a better candidate for integration into a medicinal flora than *A. mangium*, and our results clearly support this hypothesis.

*Niaouli* was rarely seen as alien or even invasive by the informants, showing that when a plant is considered useful [42], its geographical origin or ecological behavior is of little or no importance. One can suppose that for people in precarious economic and legal conditions, pragmatism rules, and that a plant which is culturally salient, abundant, and easy to recognize has a good chance to become a highly appreciated resource. Its abundance can even be seen as a blessing [68].

### Supply and distribution networks: a trade at risk?

It is quite common to come across women on the roadside, especially on weekends, picking *M. quinquenervia* and other medicinal plants for their own needs [68]. Medicinal plant trade networks, often within a single cultural group, have recently been described both within French Guiana and between French Guiana and neighboring countries, from which some diasporic groups originate [67, 69, 70]. In the case of potentially invasive species, one of the issues that these networks raise is the risk of faster distribution of these species. *A. mangium* is present in both Brazil and Suriname and *M. quinquenervia* in Suriname. If the former has a greater potential for spontaneous dispersion, the latter appears to be more restricted to places where it was intentionally introduced, from which it then spread in a slower way than *A. mangium*.

The transportation, planting or trading of these two species is illegal in French Guiana for ecological reasons. Thus, inhabitants using these plants, often already economically and legally precarious, may run the risk of prosecution. As neither the notion of invasiveness, nor the ecological issues are comprehensible to the interviewees, prosecution for using a plant would be impossible to understand and would be seen as another unfair manifestation of state overreach. Nevertheless, even if in a legal context, the uses of these plants are reprehensible, Guyanese public policies do not currently have the political will to apply repressive measures.

### Invasion or the first step of an adaptive process?

Various research has pointed out the presence of a certain cultural relativism inherent to the notion of biological invasions [44]. For the interviewees, the very idea is problematic, because all plants are useful. Considering plants as alien or invasive also reflects our representations and practices. Thus, for populations closely linked to their environment through practices of subsistence, whose interests are more local than large-scale, a plant considered by others as invasive, exotic or an ecological threat may be perceived as a new resource [71]. The arrival and expansion of a plant are not perceived as a threat to be resisted, but rather as an opportunity to be adapted to. This is the case for many species considered as invasive worldwide, which were in fact first introduced for their useful properties for humans (food, medicine, ornamentals…) or animals (fodder, medicine, fences…) [72].

The point here is not to present two opposing discourses of bioxenophobia and invaso-scepticism, but rather to take a step forward by proposing a new way of thinking about invasive species based on a case study in a particular biocultural context. In this spirit, as part of a global effort to decolonize ethnobiology [73], Tassin and Kull invite us to rethink our vocabulary and to avoid using negative metaphors based on war or disease to talk about introduced species [74]. They invite us to use a more neutral vocabulary (such as new arrivals, adventurer species, plants of change, nomad plants, voyaging plants, etc.) that does not assume that introduced species are necessarily negative disturbances that socioecosystems have to overcome.

Recent theoretical work in anthropology has challenged the notion of culture as something essentially human by pointing out the myriad interspecies dependencies [75] that underlie our systems of food, production and knowledge [29]. This recognition has led to the emergence of ‘multispecies’ approaches to anthropology [76], as well as contributed to the emerging concept of biocultural diversity [77], which describes holistic systems encompassing both human and more-than-human beings. It is also one of the starting points of the network actor theory, which postulates that non-humans are also actors [78]. From this perspective, the dynamism of cultural practices is due not only to social, political and economic factors, but also to the constantly evolving relations and relationships between humans and other beings. These complex networks of multispecies relations (often symbiotic, sometimes even co-evolutionary) reorganize during periods of intensive disruption to the system, for instance during migratory movements [33]. Alien species introductions can thus have a variety of socioecological effects, ranging from increasing agrobiodiversity to introducing species that become significant disrupters of local ecosystems. And human migration, which has always been an essential element of our ecological niche, must also be understood as a primary means of dispersal for countless animals and plants, including domestic, parasitic and other opportunistic species [79].

## Conclusion

To quote Robbins [80], “*the status and identification of any species as an invader, weed, or exotic are conditioned by cultural and political circumstances*.” Biological invasions are thus highly political. The conflicts, inconsistencies, or divisive positions that plant introduction and ecological invasion processes trigger are even, for some, a reflection of our representations, social practices, or behavioral patterns [27]. Ultimately, could not so-called ‘invasive’ plants also be seen as indicator species for both environmental and social changes [26]. Beyond the semantic debate, the emergence/establishment of these species provides new insights into our manners of understanding and managing ecological systems. Indeed, their presence requires a more integrative form of environmental governance that considers the ways in which local populations consider and respond to the arrival of new species [40, 67, 69] and tries to bridge different ethos and aesthetics (27).

This study clearly highlights some aspects of the adaptation of local ecological knowledge to new species. Recognizing new species through visual or olfactory clues, observing their ecological behavior, naming them, making hypotheses about their uses in relation to other related species (not necessarily Linnaean-related), and finally including them into daily life are quick processes that seem to be continuously ongoing, contradicting the idea of linear transmission with minimal evolution in systems of local ecological knowledge.

Finally, questioning our relationship to plant introduction may also enable us to explore our ways of conceiving migration, our approach to the otherness of life, our forms of cohabitation and our sense of hospitality.

## Supplementary Information

**Additional file 1: Appendix 2.** Informed consent form.

**Additional file 2: Appendix 1.** Questionnaire.

**Additional file 3: Appendix 3.** Table of uses de A. mangium.

**Additional file 4: Appendix 4.** Table of uses of Niaouli.

**Additional file 5:** Summary of the research in Nengee Tongo (Ndjuka Maroons language).

## Data Availability

Raw data are available on demand from the corresponding author.

## Abbreviations

IAS: Invasive alien species
UR: Use report
GEPOG: Groupe d’Etude pour la Protection des Oiseaux en Guyane
ONF: Office National des Forêts (French Forest Bureau)
CIRAD: Centre International de Recherche Agronomique pour le Développement (French International Research Center for Agronomy and Development)

## Footnotes

1 « *Dieu a créé la terre, il y a des plantes qu’on utilise, d’autres non mais chaque chose a sa place […] Si Dieu a mis toute cette verdure ici c'est qu'il y a une raison*.» Original quote translated by the authors.

2 « *Après je peux pas dire, j’ai pas envie de dire que ça a été introduit par quelqu’un. Parce que chez nous dans notre culture animiste, tout ce qui est à un endroit, c’est que c’était prédit que ça soit là. Donc dire que c’est quelqu’un qui l’a emmené là c’est comme si j’allais à l’encontre de nos croyances.*» Original quote translated by the authors.
