# Supplementary material for: Adaptive ecological knowledge among the Ndjuka Maroons of French Guiana; a case study of two ‘invasive species’: Melaleuca quinquenervia and Acacia mangium
Source: J Ethnobiol Ethnomed. 2023 Jul 11;19:29. doi: 10.1186/s13002-023-00602-7 (PMC10337182; doi:10.1186/s13002-023-00602-7)
Supplement: Supplementary file 1 — Additional file 1: Appendix 2. Informed consent form. [file 13002_2023_602_MOESM1_ESM.docx]

Appendix 2 : Informed consent form

**FORMULAIRE DE CONSENTEMENT ECLAIRE**

**Notice explicative**

Dans le cadre de son stage de master 2 à l'Université de Montpellier, la stagiaire Johanna Theys va s'intéresser aux usages, représentations et savoirs naturalistes locaux des espèces considérées invasives que sont l'*Acacia mangium* et le Niaouli (*Melaleucaquinquenervia*). Ces travaux seront menés essentiellement dans l'ouest Guianais, dans les zones de savanes littorales, via des entretiens, et viseront à décrire les rapports qu'entretiennent les habitants de ces régions avec les deux espèces mentionnées.

Dans ce cadre, la stagiaire vous interrogera sur vos savoirs, pratiques et perceptions, de manière anonyme. Vous êtes invités à y répondre, sans aucune obligation. Il ne sera fait aucun usage commercial, ni des données personnelles de ces questionnaires, ni des savoirs recueillis dans le cadre de ce stage.

Pour toute information, vous pouvez contacter les encadrants de ce stage : au GEPOG (Groupe d'Etude pour la Protection des Oiseaux de Guyane), Mme Alizée Ricardo (alizee.ricardou@gepog.org), au CNRS, Mr Guillaume Odonne (guillaume.odonne@cnrs.fr), ou la stagiaire elle-même (johannatheys@hotmail.com).

**Consentement en connaissance de cause**

*Après avoir discuté et obtenu réponses à mes questions, j’accepte librement et volontairement de participer à la recherche décrite ci-dessus. Je suis parfaitement conscient(e) que je peux retirer à tout moment mon consentement sur ma participation à cette recherche quelles que soient mes raisons. Le fait de ne plus participer à cette recherche ne portera pas atteinte à mes relations avec l’enquêteur.*

Enquêteur

NOM, Prénom :

Date :

Signature :

Personne donnant son consentement

NOM, Prénom :

Date :

Signature :
