# Supplementary material for: Adaptive ecological knowledge among the Ndjuka Maroons of French Guiana; a case study of two ‘invasive species’: Melaleuca quinquenervia and Acacia mangium
Source: J Ethnobiol Ethnomed. 2023 Jul 11;19:29. doi: 10.1186/s13002-023-00602-7 (PMC10337182; doi:10.1186/s13002-023-00602-7)
Supplement: Supplementary file 2 — Additional file 2: Appendix 1. Questionnaire. [file 13002_2023_602_MOESM2_ESM.docx]

**Appendix 1 : Questionnaire**

Numéro d'entretien : ............................................................

Date de l'entretien : ..............................................................

Lieu de l'entretien : ...............................................................

*Dans le cadre de mon stage de master 2 à l'Université, je m’intéresse aux usages, représentations et savoirs locaux à propos de deux espèces de plantes. Pour comprendre ça, je vais interroger des habitants sur ce qu’ils savent sur ces espèces. Je vais essentiellement travailler dans l’ouest de la Guyane, dans les zones de savanes littorales, et dans les endroits où ces espèces poussent.*

*Est-ce que vous seriez d’accord pour répondre à quelques questions afin de m’aider à réaliser mon étude ?*

*Comme je le disais, je vais vous interroger sur* ***vos savoirs, pratiques et perceptions****, de manière* ***anonyme****. Vous pouvez y répondre, sans aucune obligation. Il ne sera fait* ***aucun usage commercial, ni des données personnelles de ces questionnaires, ni des savoirs recueillis dans le cadre de ce stage.***

1. **Donnéespersonnelles**
   1. Est-ce que vous pouvez-vous présenter un peu ? (expliquez pourquoi il s’agit d’informations importantes pour nous)

(Si pas répondu dans la présentation, demandez) :

- - 1. Quel âgeavez-vous ?
    2. Où est-ce que vous êtes né.e ?
    3. Quand et comment êtes-vous venus vivre ici ?
    4. Avec qui vivez-vous ?
    5. Quelleslanguesparlez-vous ?
    6. Quelle a été la première langue que vous avez apprise ? (langue de socialisation)
    7. Est-ce que vous avez été à l’école ? Est-ce que vous êtes allé.e au collège ? au lycée ? avez-vous continué ensuite ? si oui, par quoi ?
    8. Comment occupez-vous vos journées ? (travail, profession)
    9. Est-ce que vous avez toujours fait ça ou faisiez-vous autre chose auparavant ?
    10. Avez-vousunereligion ?

1. **Savoirsnaturalisteslocaux**

*Après avoir montré les planches de photographies et les parties de plantes fraîches ou séchées*

- 1. Connaissez-vouscetteplante ?
  2. Comment l’appelez-vous ?
  3. Est-ce que vous utilisez d’autres noms ? Est-ce que vous connaissez d’autres noms ? Est-ce que vous avez entendu des gens l’appeler autrement ?
  4. Où trouvez-vous/ observez-vous cette plante ?

| Jardin de la maison |  |
| --- | --- |
| Bords de route |  |
| Abattis, parcelleagricole |  |
| Savanes |  |
| Ailleurs/autre |  |
| Jamais vu |  |

- 1. Est-ce que vous avez déjà vu cette espèce ailleurs ? (sioui, précisezoù)

| Dans d’autres endroits où vous avez habité |  |
| --- | --- |
| Dans d’autres endroits de Guyane |  |
| En dehors de la Guyane/ à l’étranger |  |

- 1. Est-ce qu’il s’agit d’une espèce que vous voyez souvent ?

| Tous les jours |  |
| --- | --- |
| De temps en temps (si c’est le cas, quand est-ce que vous l’avez-vu pour la dernière fois ?) |  |
| Jamais |  |

- 1. Avez-vous toujours connu cette espèce ou l’avez-vous découverte récemment ? Est-ce que vous vous souvenez de la première fois où vous l’avez vu ?Est-ce que vous avez remarqué des choses sur cette espèce ? (Par exemple…)

| Vitesse de croissance : *à quelle vitesse est-ce qu’elle grandit ?* |  |
| --- | --- |
| Méthode de dispersion : *est-ce qu’elle fait beaucoup de graines ? pas beaucoup de graines ? Comment elle se disperse ?* |  |
| Floraison : *quand est-ce qu’elle fleurit ? Quelle couleur est la fleur ?* |  |
| Zones de pousse : *où est-ce qu’elle se met à pousser ?* |  |
| Espèces proches : *est-ce qu’elle ressemble à d’autres plantes que vous connaissez ?* |  |

1. **Usages**
   1. Est-ce que vous utilisez cette plante ?
   2. A quoi vous sert-elle ?(Demande de description de l’usage : comment faîtes-vous ?)
   3. Quelles parties de la plante utilisez-vous ?

| Feuilles |  |
| --- | --- |
| Ecorce |  |
| Bois |  |
| Fleurs |  |
| Graines/gousses |  |
| Racines |  |

- 1. Qui vous a appris à l'utiliser ?
  2. Est-ce que vous/ les gens utilise cette plante depuis longtemps ?
  3. Comment vous la procurez-vous ?

|  | Lieu | Avec qui |
| --- | --- | --- |
| Achat |  |  |
| Culture/ Plantation |  |  |
| Cueillette |  |  |
| Troc/don |  |  |

- 1. Est-ce que vous avez vous-même déjà vendu cette plante ?
  2. Est-ce que vous connaissez d'autres usages ? Si oui, lesquels ?

1. **Représentations, perceptions**
   1. Est-ce que vous pensez que cette plante a toujours été en Guyane ou est-elle arrivée récemment ? (Si deuxième réponse : comment est- elle arrivée en Guyane selon vous ?)
   2. Que pensez-vous de cette plante ?

|  | Pourquoi ? |
| --- | --- |
| Positif |  |
| Négatif |  |
| Neutre |  |

- 1. Est-ce que vous avez l’impression qu’on trouve cette plante plus qu’avant ? Dans de nouveaux endroits ?
  2. Est-ce que cette plante vous a déjà posé problème ou vous pose problème actuellement ?
  3. Est-ce que vous avez déjà eu besoin de vous débarrasser de cette plante ?
  4. Si oui, comment vous en êtes-vous débarrassé ?

| Coupe |  |
| --- | --- |
| Arrachage manuel des jeunes pousses |  |
| Feu |  |
| Autre (précisez) |  |

- 1. Est-ce que cela a été efficace ?
  2. Est-ce que vous pensez qu’il est faudrait que cette plante disparaisse entièrement du territoire Guianais ?
  3. Pourquoi ?
  4. Connaissez-vous le terme “espèce invasive” ? Qu’est-ce que cela signifie pour vous ?
  5. Les associations de protection de l'environnement souhaitent faire disparaître cette plante car elles la considèrent néfaste, parce qu’elle envahit les savanes. Qu’enpensez-vous ?
