# Supplementary material for: Adaptive ecological knowledge among the Ndjuka Maroons of French Guiana; a case study of two ‘invasive species’: Melaleuca quinquenervia and Acacia mangium
Source: J Ethnobiol Ethnomed. 2023 Jul 11;19:29. doi: 10.1186/s13002-023-00602-7 (PMC10337182; doi:10.1186/s13002-023-00602-7)
Supplement: Supplementary file 3 — Additional file 3: Appendix 3. Table of uses de A. mangium. [file 13002_2023_602_MOESM3_ESM.docx]

Appendix 3 : Table of uses de A. mangium

| **Use Category** | **Usage** | **URs** | **Quotes** |
| --- | --- | --- | --- |
| MUT | Charcoal | 9 | *« Charbon pou nous, pou lé grillades »* (entretien 5, haï)  “It’s charcoal for us, to use for grilling” |
| MUT | Firewood (for cooking) | 5 | *« On l'aime beaucoup pour faire le feu, il brûle bien, ça prend le feu vite quand il est sec, pour faire le kwak »* (entretien 24, bus) ; « *C'est bien pour le feu, pour faire coffee, faire manger. »*(entretien 26, bus)  “We like it a lot to make fires, it burns well, when it’s dry it catches fire quickly, we use it to make kwak”  “It’s good for fire, to make coffee, to cook food” |
| MMRC | Treating fever, in particular for body aches linked to fever | 4 | *« Je crois que mon père l’a déjà utilisé pour nous, pour faire des bains. [...] Par exemple, si l’un de nous a de la fièvre, il les cueille pour nous, pour nous aider à nous soigner, on fait des bains avec, parfois on les boit, mais il le mélange avec d’autres plantes. »* (entretien 54, bus) ; « *C’est un bain pour quand tu as des courbatures. »* (entretien 55, bus)  “I believe that my father used it for use, to give baths…for example, if one of us had a fever, he would harvest it to help to heal us, we would take baths with it, sometimes drink the tea, but he would mix it with other plants”  “It’s for baths when you have body aches” |
| MUT | Fertilizer (nitrogen) | 3 | *« Ça apporte énormément azote à la terre […] si tu as ça sur ta parcelle, il ne t’oblige plus à mettre un engrais vert à chaque rotation parce que tu as ça sur la parcelle »* (entretien 60, bus)  “It brings a huge amount of nitrogen to the soil…if you have this on your land, you don’t need to add fertilizer each growing season because you have this tree on your parcel.” |
| MUT | Timber | 2 | *« C’est un bon bois si c’est grand : le cœur est dur. »* (entretien 60, bus)  “It’s a good wood if the tree is big: the heartwood is hard.” |
| MMRC | Body care | 2 | *« Bon pour laver »* (entretien 33, bus) ; *Non on s’en sert pas trop, on l’utilise en bain quand on a par exemple, mon père n’a pas été pour chercher les feuilles qu’on utilise d’habitude […] voilà, pour ne pas rester sans rien on l’utilise. »* (entretien 29, bus)  “It’s good to wash with.”  “No, we don’t use it too much, but we use it for baths for example when my father hasn’t gotten the leaves we usually use…so we aren’t left with nothing, we use it.” |
| MMRC | Sexuality | 2 | *« Pour laver la chouchoune, c’est plus fort encore »* (entretien 48, bus)  “To wash the vulva, it’s even stronger” |
| MMRC | Treatment of Covid-19 | 2 |  |
| MUT | Compost (to speed up decomposition) | 1 | *« Moi je couvre toujours mon compost avec les feuilles, j’ai remarqué ça dégrade beaucoup plus vite qu’avec les autres feuilles qu’on couvre, avec les feuilles de banane […] »* (entretien 60, bus)  “I always cover my compost with the leaves. I noticed that it breaks down much faster than the other leaves that we use, for example banana leaves…” |
| MUT | Wood chips as soil improver | 1 | *« Chez moi tu vois là, ça pousse partout, mais chez moi c’est fait exprès […] comme ça je vais pas loin dans la forêt pour couper les repousses de bois pour broyer. »* (entretien 60, bus)  “We have it at my house. It grows everywhere, but at my house we grow it on purpose…this way I don’t have to go far into the forest to cut the young branches and grind it up” |
| MUT | Mulch | 1 | *« Les feuilles tu peux utiliser comme la couverture du sol. »* (entretien 60, bus)  “You can use the leaves as mulch” |
| MUT | Construction of poles and pestles | 1 |  |
| MMRC | Treatment of flu | 1 |  |
| MMRC | Diuretic | 1 | *« […] pour se rafraîchir et évacuer beaucoup de toxines parce que quand on le boit en thé on fait beaucoup pipi. »* (entretien 55, bus)  “…to refresh yourself and to cleanse a lot of toxins, because when you drink it in tea, you pee a lot” |
| MMRC | Treatment of back pain | 1 |  |
| MMRC | Treatment of skin problems | 1 |  |
| MMRC | Treatment of ‘cold’ | 1 |  |

MMRC: Medicinal, Magico-Religious and Cosmetic ; MUT: Material and Technical Usages; Bus: Person of bushinenge origin; Haï: person of Haitian origin
