# Supplementary material for: Adaptive ecological knowledge among the Ndjuka Maroons of French Guiana; a case study of two ‘invasive species’: Melaleuca quinquenervia and Acacia mangium
Source: J Ethnobiol Ethnomed. 2023 Jul 11;19:29. doi: 10.1186/s13002-023-00602-7 (PMC10337182; doi:10.1186/s13002-023-00602-7)
Supplement: Supplementary file 4 — Additional file 4: Appendix 4. Table of uses of Niaouli. [file 13002_2023_602_MOESM4_ESM.docx]

Appendix 4 : Table of uses of Niaouli

| **Use Category** | **Usage** | **URs** | **Quotes** |
| --- | --- | --- | --- |
| MMRC | Vaginal steam bath | 32 | *« Ça c’est pour les femmes »* (entretien 15, bus) ; *« Si tu laves avec ça, ton mari il va jamais partir »* (entretien 59, bus) ; *« Ca serre et ça bouche tout comme si t’étais vierge. […] Tu t’assoies là sur ton truc, c’est chaud et tu t’assoies et ton ventre ça te fais mal et ça te calme petit à petit. Oui ça t’aide parce que ça réchauffe ton ventre si t’es froid dedans et puis tu bois le bita, c’est parfait et tu te retrouves. »* (entretien 56, bus)  “This is for women.”  “If you wash with it, your husband will never leave you.”  “It tightens and closes everything up just like you are a virgin…you sit down on your bucket, it’s hot and you sit down and if your belly hurts, this will calm it little by little. Yes, it helps you because it warms up your belly if you are cold inside, and then you drink bitters, it’s perfect and you get well again.” |
| MMRC | Treatment of fever (*feba*), notably flu | 18 | *« Quand on a frette ou grippe c’est bon »* (entretien 21, bus)  “It’s good when we have the cold or the flu.” |
| MMRC | Post-partum care | 17 | *« Il faut laver 3 mois après accouchement avec Albina uman et d’autres, y a d’autres, on mélange tout »* (entretien 49, bus) ; *"Oui, pour son odeur et le Fekisi uwii, c’est vraiment quelque chose qu’on utilise après l’accouchement, parce qu’en fait quand on a accouché, on a cette sensation d’avoir des odeurs par rapport aux liquides qu’on va évacuer, du coup on utilise celle-là parce que ça a une odeur de Vicks"*(entretien 29, bus)  “You have to wash three months after giving birth with *Albina uman* and other plants, there are others, we mix them all.”  “Yes, for its smell, the *Fekisi uwii* is really something that we use after giving birth, because in fact when we have given birth, we have this sense of having bad smells because of the fluids that we have expelled, so we use this because it smells like Vicks.” |
| MMRC | Treatment of sick infants | 11 | *« Et ça guérit hein, quand ton bébé malade tout ça. »* (entretien 60, bus)  “It heals well, when your baby is sick and all that.” |
| MMRC | Treatment of covid-19 | 5 | *« Tu sais j’utilise pas qu’une fois hein, j’utilise plusieurs fois, à chaque fois que j’ai la fièvre, avec le covid aussi »* (entretien 20)  “You know I haven’t just used it one time, I’ve used it many times, each time that I had the fever, and with covid as well.” |
| AL | Infusion de confort | 4 | *« Ça c’est ma grand-mère qui faisait des thés avec ça. Et y met ça dans lait aussi. Hmhm. Quand on fait des chocolats y met ça aussi. Pour aromatiser. »* (entretien 57, haï)  “It was my grandmother than made tea with this. And she also put it in milk. Hmm. When we made chocolates she would put it in as well, to make it smell good.” |
| AL | Cleaningfish or meat | 4 | *« On peut cuisiner avec, si la viande que tu as chassée ne sent pas bon pour toi, tu laves avec et tu rinces, après c’est mieux. »*(entretien 27, bus)  “We can cook with it. If the meat that you hunted doesn’t smell good to you, you clean it with the niaouli and rinse it, and then it’s better” |
| MMRC | Weight loss | 3 | *« Ça fait maigrir mais pas pour toute les femmes »* (entretien 49, bus)  “It makes you lose weight, but it doesn’t work for all women.” |
| MMRC | Treatment of respiratory problems | 3 | *“Mi si enke a fekisi uwii sa abi taa bun aini a deesi, gi ala den deesi di abi fekisi a ini te ju luku na a sumee fu en gi boo fu ati anga ala den sani fu a sikin ini di e jeepi fu boo.”* (entretien 63 bus)  “She thinks that niaouli, because of its smell, can have medicinal effects for the respiratory system like any other mentholated remedy.” |
| AL | Spice | 3 |  |
| MMRC | Magico-religious use | 2 |  |
| MMRC | Treatment of tooth problems | 2 | *« Si tu as une carie, tu mets ça dans la bouche. »*(entretien 30, bus)  “If you have a cavity, you put this in your mouth” |
| MMRC | Body care | 2 |  |
| MMRC | Prevention of covid-19 | 2 | *« Oui mais en prévention comme je dis, c’est pas non plus une odeur aussi forte que le Vicks du genre tu vas l’utiliser il t’arrivera plus rien. […] C’est avant. Si tu l’utilise pendant que t’es malade, ça va pas faire l’effet doliprane. […] C’était en prévention, pendant que tu l’as ça continue à agir parce que t’avais commencé en prévention, donc tu continues mais si tu le prends parce que t’es malade c’est sûr que ça fait rien pour toi. C’est pas une plante super concentrée non plus quoi. »* (entretien 29, bus)  “Yes but it is for prevention, as I said, it’s not as strong a smell as Vicks, like if you use it nothing will happen…it’s for before. If you use it when you are sick, it will not kill the pain…it is for prevention, but when you have covid, it will continue to act because you started using it preventatively, then you continue, but if you take it because you are sick, it’s certain it won’t do anything for you. It’s not a very strong plant.” |
| MMRC | Treatment of insect bites | 1 |  |
| MMRC | Treatment of stomachache | 1 |  |
| MMRC | Antibacterial | 1 |  |
| MUT | Firestarter | 1 |  |
| MMRC | Anti-mosquito | 1 |  |
| MUT | Substrate for plants | 1 |  |
| MUT | Keeping the soil moist | 1 |  |
| AL | Tonic | 1 | *“La feuille tu fais bouillir, après tu mets dans le rhum."*(entretien 53, bus)  “You boil the leaves and then put them in a bottle of rum” |

MMRC: Medicinal, Magico-Religious and Cosmetic; MUT: Material and Technical Usages; AL: Alimentary; Bus: Person of bushinenge origin; Haï: person of Haitian origin
