# Supplementary material for: Adaptive ecological knowledge among the Ndjuka Maroons of French Guiana; a case study of two ‘invasive species’: Melaleuca quinquenervia and Acacia mangium
Source: J Ethnobiol Ethnomed. 2023 Jul 11;19:29. doi: 10.1186/s13002-023-00602-7 (PMC10337182; doi:10.1186/s13002-023-00602-7)
Supplement: Supplementary file 5 — Additional file 5: Summary of the research in Nengee Tongo (Ndjuka Maroons language). [file 13002_2023_602_MOESM5_ESM.docx]

**A toli moo satu**

**A hii toli:** Fu man fustan fa a sabi anga a bun tan fu wi gaan sama sani e kengi anga fa wi e tan anga den, te njunjun sani (uwii, bon) komoto fu taa kondee kon na wi kondee. Wi wani tjai den sabi anga a pakisei fu den busikondee sama fu ‘Laguyane ’kon na fesi abaa tu bon. Wan fu den na *Acacia mangium* a tawan na Niaouli (*Melaleuca quinquenervia*). Den tu bon ja, leliman e si den enke “uwii efu bon di e poli peesi”a ini den sabana anga den peesi pe sama e tan.

**A fasi fa wi wooko**: Fu a wooko ja, wi piki piki a pakisei fu wan tu sama a ini a pisi fu fo mun fu baka jali go miti seibin mun fu baka yali. Wan duupu falaki akisi be meke poti a pampila fu wi go akisi den sama. Wan tu pisi fu den bon wi be teke, fowtow fu den be puufu du a wooko. Fa den e wooko anga den uwii, den sabi di den sama fu a pisi wataa ja abi a den uwii ja tapu, fa den e si den, dati be de a wooko anga a ondoosuku di wi meke anga den busikondee sama fu ‘Laguyane’ na hii se fu soolan pisi anda. Ala san den sama soi wi, naki a masini fu wisa teli den.

**San wi fende**: A e soi taki den sama fu a kondee ja e teke den uwii a ini den sabi, a ini den aladei libi. Den gi den nen, den e koboloiki den, den e seli den. Da te wi luku fini da wi si taki den uwii ja no gei sani di komoto a taa faawe kondee kon dise efu a na e gei enke den e si en enke uwii di e poli peesi efu di e gi den polobelema. Da a jeepi fu den uwii ja de wan gaan sani a ini den busideesi (osudeesi) fu den busikondee sama anga tu fa a sabi fu den e kai anga njun uwii di kon.

**A tapun**: Boiti fu a soi di wi wani soi fa a de taanga fanowdu fu tjai a taki anga pakisei fu den sama ja kon na fesi a ini a seeka fu a kondee abaa den tu uwii ja, di e soi enke uwii di e gi polobelema. A ondoosuku ja e soi wan fasi fu wooko anga njun uwii di kon, te doo na den seefi enke libi sama di lowe kon enke njun sama a ini a kondee di den a be sabi.

**San wi fende**: A ondoosuku ja e soi taki a njun fasi fu teke leli anga njun sabi fu njun uwii sa e pasa moo fuu anga moo esi esi.

**Den moo gaan wowtu**: Bon (uwii) di e poli peesi, busikondee sama (malon), den sabi fu wi pisi kondee, kengi fasi fu libi, fu pakisei anga taa kondee sama, a fasi fu si a goon mama.
